# The impact of visuospatial perception on distance judgment and depth perception in an Augmented Reality environment in patients after stroke: an exploratory study

**DOI:** 10.1186/s12984-021-00920-5

**Published:** 2021-08-21

**Authors:** Chiara Höhler, Nils David Rasamoel, Nina Rohrbach, John Paulin Hansen, Klaus Jahn, Joachim Hermsdörfer, Carmen Krewer

**Affiliations:** 1 Technical University of Munich, Georg-Brauchle Ring 60/62, 80992 Munich, Germany; 2 Technical University of Denmark, Anker Engelunds Vej 1, 2800 Kgs. Lyngby, Denmark; 3 Schoen Clinic Bad Aibling, Kolbermoorer Strasse 72, 83043 Bad Aibling, Germany; 4 Ludwig-Maximilians University of Munich, University Hospital Grosshadern, Marchioninistrasse 15, 81377 Munich, Germany

**Keywords:** Depth perception, Distance perception, Visual perception, Augmented Reality, Stroke, Virtual systems

## Abstract

**Background:**

Augmented Reality (AR)-based interventions are applied in neurorehabilitation with increasing frequency. Depth perception is required for the intended interaction within AR environments. Until now, however, it is unclear whether patients after stroke with impaired visuospatial perception (VSP) are able to perceive depth in the AR environment.

**Methods:**

Different aspects of VSP (stereovision and spatial localization/visuoconstruction) were assessed in 20 patients after stroke (mean age: 64 ± 14 years) and 20 healthy subjects (HS, mean age: 28 ± 8 years) using clinical tests. The group of HS was recruited to assess the validity of the developed AR tasks in testing stereovision. To measure perception of holographic objects, three distance judgment tasks and one three-dimensionality task were designed. The effect of impaired stereovision on performance in each AR task was analyzed. AR task performance was modeled by aspects of VSP using separate regression analyses for HS and for patients.

**Results:**

In HS, stereovision had a significant effect on the performance in all AR distance judgment tasks (*p* = 0.021, *p* = 0.002, *p* = 0.046) and in the three-dimensionality task (*p* = 0.003). Individual quality of stereovision significantly predicted the accuracy in each distance judgment task and was highly related to the ability to perceive holograms as three-dimensional (*p* = 0.001). In stroke-survivors, impaired stereovision had a specific deterioration effect on only one distance judgment task (*p* = 0.042), whereas the three-dimensionality task was unaffected (*p* = 0.317). Regression analyses confirmed a lacking impact of patients’ quality of stereovision on AR task performance, while spatial localization/visuoconstruction significantly prognosticated the accuracy in distance estimation of geometric objects in two AR tasks.

**Conclusion:**

Impairments in VSP reduce the ability to estimate distance and to perceive three-dimensionality in an AR environment. While stereovision is key for task performance in HS, spatial localization/visuoconstruction is predominant in patients. Since impairments in VSP are present after stroke, these findings might be crucial when AR is applied for neurorehabilitative treatment. In order to maximize the therapy outcome, the design of AR games should be adapted to patients’ impaired VSP.

*Trial registration:* The trial was not registered, as it was an observational study.

**Supplementary Information:**

The online version contains supplementary material available at 10.1186/s12984-021-00920-5.

## Background

Visuospatial perception (VSP) is the ability to appropriately perceive the physical location of an object in relation to the own body and to identify the physical relationship between different objects. Concretely, VSP describes the interpretation of size, shape, position and motion of objects [1]. Visuospatial abilities are mandatory for functional tasks in everyday life [2], such as buttoning a shirt, assembling furniture [3], grasping objects and evaluating distances in road traffic [4]. VSP involves several aspects, such as visuoconstruction, binocular stereopsis and spatial localization [5]. Visuoconstruction is the ability to see a picture in its single elements and to re-arrange them to the original picture with respect to their spatial relationships [3]. Binocular stereovision describes the perception of depth. It is defined as the ability to visually perceive the world as three-dimensional (3D) and to estimate the distance of an object [6]. Although the retina of our eyes perceives the world as a flat and two-dimensional (2D) image, the fusion of both retinal images leads to the generation of a 3D image in the occipital cortex [7]. Spatial localization abilities are involved in the discrimination of shapes and their localization in space [5].

The processing pathway of visuospatial information starts with the projection of the visual field on the retinas of both eyes. Photoreceptors of both retinas transfer the images of each eye separately as neural signals via the optic nerves. Both optic nerves meet each other at the optic chiasm, where the nasal fibers cross over. Visual information is further transmitted over the corpus geniculatum laterale to the occipital cortex [8]. This is the first cortical area that receives and fuses visual input from both eyes. Corresponding retinal points are interpreted in the occipital cortex, for which intact communication of the right and left brain hemisphere through the corpus callosum is essential [9]. Cortical information processing continues in higher areas of the cortical visual system over two separate processing streams: the dorsal and the ventral occipital pathways [10]. Specifically, neurons of the dorsal pathway, which proceeds from the occipital cortex over V2 and MT/V5 to the intraparietal area of the brain [10], process visuospatial information and are sensitive for disparity. On a high visual processing level, the neurons of the dorsal pathway are involved in the evaluation of depth, motion and location of objects [11], as well as in the decision on how to use, manipulate and interpret objects [12]. The path of the ventral occipital stream starts in V1, proceeds over V2 and V4, and ends in the inferiortemporal area [10]. In the ventral pathway, neurons are sensitive for the detection of object properties. Neurons in the inferiortemporal area are for example responsible for size constancy. The size of a familiar object is interpreted as being the same, independent of the object’s distance. Even though the object projects different absolute sizes on the retina with varying distance, the perception of the object’s size remains stable [8].

After a stroke, where the brain tissue is damaged due to a lack in oxygen supply [13], the above-mentioned neural structures are commonly impaired in survivors, which has an impact on VSP. The most prevalent impairment in VSP in patients after stroke is spatial neglect [14], where the patient shows difficulties in orientation and attention in the contralesional space [15]. Secondly, hemianopia which is the loss of visual perception in one hemifield is a common symptom after stroke leading to impairments in VSP [9]. Thirdly, every fifth patient after stroke is classified as being stereoblind [16]. Besides perceptual difficulties, stroke survivors are affected by eye movement disorders, like gaze palsy, heterotropia (in the reference paper [17] the term strabismus is used) and nystagmus [17]. Eye movement disorders hinder the eyes in giving an adequate input to the occipital cortex and lead to deficits in stereopsis and to visuospatial impairments, since the integration of binocular information in the occipital cortex is impaired [9, 18].

Independent of the fact that patients after a stroke show deficits in VSP, Augmented Reality (AR), Virtual Reality (VR) and active video games are used as technological interventions in the setting of neurorehabilitation [19–23]. These technological interventions have the potential to positively affect motor learning, as patients enjoy the training sessions, show engagement in the therapy and are motivated [19, 20]. Some patients are additionally encouraged by the challenging character of the games and the possibility of receiving real-time feedback on their performance [21]. AR, VR and gaming devices provide the opportunity to easily adapt the task difficulty to the patient’s individual capabilities and thus make individualized treatment possible [22]. Furthermore, these technological interventions can address cognitive functions such as memory [21] and also support patients with cognitive impairments in the performance of Activities of Daily Living (ADL [24]). Another advantage of AR systems in particular, where the real environment is augmented in real-time by interactive, virtual 3D holographic objects [25], is the possibility to perform ADL in a more functional way that is similar to typical daily activities [26]. Intervention studies, which investigate the effectiveness of AR applications in combination with robotic devices for the upper limb (UL) rehabilitation in patients after stroke, have found improvements in the UL functions after the intervention [23]. However, in interventions like these, the patient reaches and moves holographic objects that are placed at different distances from the user. This indicates that the individual’s VSP is crucial for the intended interaction in the AR environment and thus for benefitting from the AR intervention [27].

Previous findings from literature including healthy participants indicate that AR devices are able to provide stereoscopic images to the user and conclusively give an instant understanding of depth in the AR environment [27–29]. According to the subjective perception of holographic objects of patients after stroke, their responses are inconsistent regarding the appearance of holograms as (1) placed in the room, (2) touchable and reachable, and (3) as real or natural objects [30]. Nevertheless, it has not been investigated whether deficits in VSP after stroke are associated with the perception of holographic objects. Still, it is unclear if patients with impairments in VSP are able to perceive virtual holograms as 3D and to adequately judge distance in the AR environment.

To address this gap in literature, the ‘SPiAR—Spatial Perception in Augmented Reality’ study was initiated. To compare impairments in VSP with depth perception during an AR application, stereoscopic vision and spatial perception in a real surrounding and in an AR environment were assessed in patients post stroke and in healthy subjects (HS). The primary aim was to compare the perception of holographic objects between subjects with and without impairments in stereovision. The secondary aim was to investigate the relationship between VSP in the real environment and VSP perception of virtual holograms in the AR environment.

## Methods

### Study design

The purpose of this study was to investigate the impact of impairments in VSP on the ability to perceive depth during an AR application in patients after stroke. In the design of an exploratory study, stereovision and spatial perception in the real and the AR environment were assessed. In the first step, a group of HS served to test the validity of the newly developed AR tasks in assessing stereovision. In the second step, patients after stroke were tested. Every participant performed the testing procedure once in a 40-min session. Patients with attentional or cognitive deficits participated in two separate sessions with a duration of 20 min each.

### Participants

**Fig. 1:**
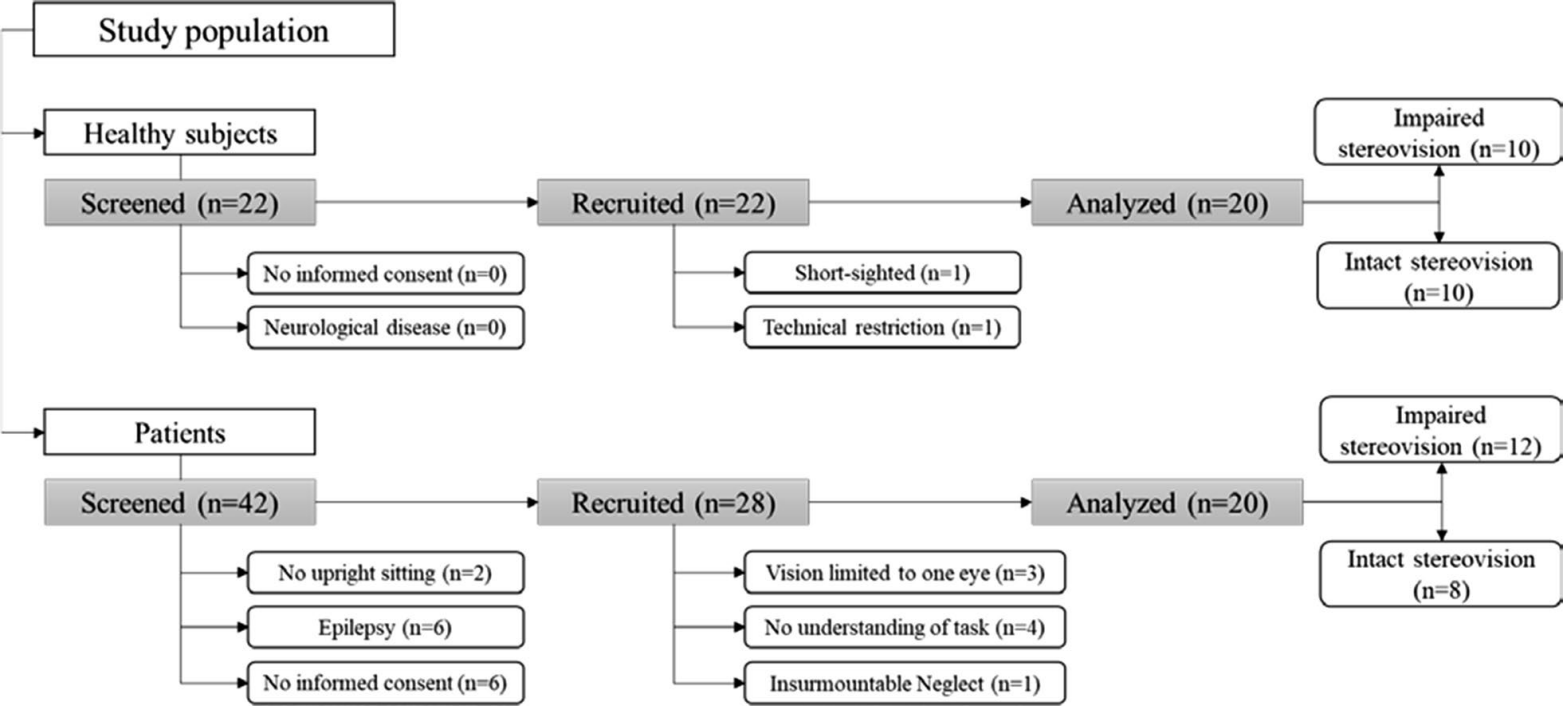
The study population of the SPiAR study

As the incentive behind this study was to improve the applicability of AR treatment in neurorehabilitation, potential users of AR therapy were addressed. Consequently, patients who received either robotic or AR treatment were screened for eligibility to participate (n = 42). Inclusion criteria was the diagnosis of a stroke, without restriction of the location of the lesion, since it is unclear whether or not the site of the stroke affects depth perception [31]. HS were recruited by word of mouth. Ten subjects with normal or corrected to normal vision and ten individuals showing problems in stereovision were recruited. These two subgroups of HS were matched for sex and age (± 2 years). Exclusion criteria for patients and HS are presented in Fig. 1.

The study was approved by the Ethics Commission of the Medical Faculty of the Technical University of Munich (registration number: 175/17 S) and conducted according to the principles of good clinical practice. All participants provided written informed consent prior to the testing procedure.

### Clinical tests

Different aspects of VSP were assessed: stereovision and spatial localization/visuoconstruction.

Stereovision in real world was tested using two standard clinical assessments: the *Titmus-Test* (Stereo Optical Co., Chicago, IL) and the *Lang II Stereotest* (LANG-STEREOTEST AG, Küsnacht, Switzerland). The *Titmus-Te*st consists of two subtests measuring the presence of stereovision (*Stereo-Fly Test*) and the quality of stereovision (*Circles* test). Both subtests are performed at a distance of 40 cm away from the subject, while wearing polarized glasses (45°/135°) that produce vectograph images. In the *Stereo-Fly Test*, a fly is presented to the subject with a disparity of 3552 s of arc (''). Participants were asked to touch the fly’s wings with a pinch movement of the index finger and the thumb. Correct test performance and thus intact stereovision is given when the subject pinches the wings, which are floating in the air above the board. Quality of stereovision was measured by means of the *Circles* test, where different levels of stereoacuity are presented. Nine diamond shaped clusters consisting of a set of four vectographic circles were presented to the participant. In every diamond one circle is depicted with a disparity ranging from 800'' to 40'' and thus gives the impression of standing in front of the other circles, when wearing the polarized glasses [32]. The difficulty increases with progressing from one cluster to the next, as the degree of disparity decreases. The outcome variable of the *Circles* test was the detected stereoacuity level in seconds of arc in the last correctly named cluster. For statistical analysis, the stereoacuity level was transformed to categories from zero to ten (0 ≙ no stereoacuity level was detected; 1 ≙ 5335″; 2 ≙ 800″; 3 ≙ 400″, 4 ≙ 200″, 5 ≙ 140″; 6 ≙ 100″; 7 ≙ 80″; 8 ≙ 60″; 9 ≙ 50″; 10 ≙ 40″).

The *Lang II Stereotest* requires the subject to identify an object that is concealed in a Random Dot Stereogram (RDS). One stimulus card, presenting four different disparity-defined objects, is presented at a distance of 40 cm to the person. Three of these concealed objects (elephant, truck and moon) are presented at different disparity levels (600″, 400″ and 200″) in a RDS. The fourth object (star) can be detected with monocular vision. Tests that are based on a RDS, like the *Lang II Stereotest*, assess global stereopsis. The outcome of the *Lang II Stereotest* was either ‘pass’ or ‘failure’. There were three scenarios in which the subject passed the test: (1) all three binocular objects were named correctly, (2) depth was appreciated in all three binocular objects, but the shape was not named, or (3) two out of three binocular objects were correctly named and the negative response was at lowest stereoacuity level (moon). All other scenarios were recorded as ‘failure’ [33].

Spatial localization/visuoconstruction was tested using the *Visual Object and Space Perception (VOSP)* test battery [34]. This test battery includes four validated and standardized tasks, namely (1) Dot Counting, (2) Position Discrimination, (3) Number Location, and (4) Cube Analysis [34, 35]. For a successful performance of the VOSP test battery, intact spatial processing and visuoconstructional abilities are required in order to scan the stimulus card, localize objects, and interpret their relative position [36]. In the Dot Counting task the participant named the number of randomly arranged dots on ten stimulus cards. Twenty stimulus cards were presented in the Position Discrimination task, each showing two squares next to each other, one with a centered and one with an not-centered dot. Participants were instructed to name the square, in which the dot is centered. The Number Location task consisted of ten cards presenting two squares above each other. The upper square showed randomly placed numbers, the lower one a dot which was located in the same position as one of the numbers above. The instruction of this test was to name the number in the upper square that is located in the same position as the dot. Ten stimulus cards, which show 2D drawings of a 3D configuration of cubes were presented in the Cube Analysis. Subjects interpreted how many cubes the drawing on the stimulus card contains. Outcome of the VOSP test battery was the number of correctly answered stimulus cards with a maximum score of 50 points.

### Tests in the AR environment

**Fig. 2:**
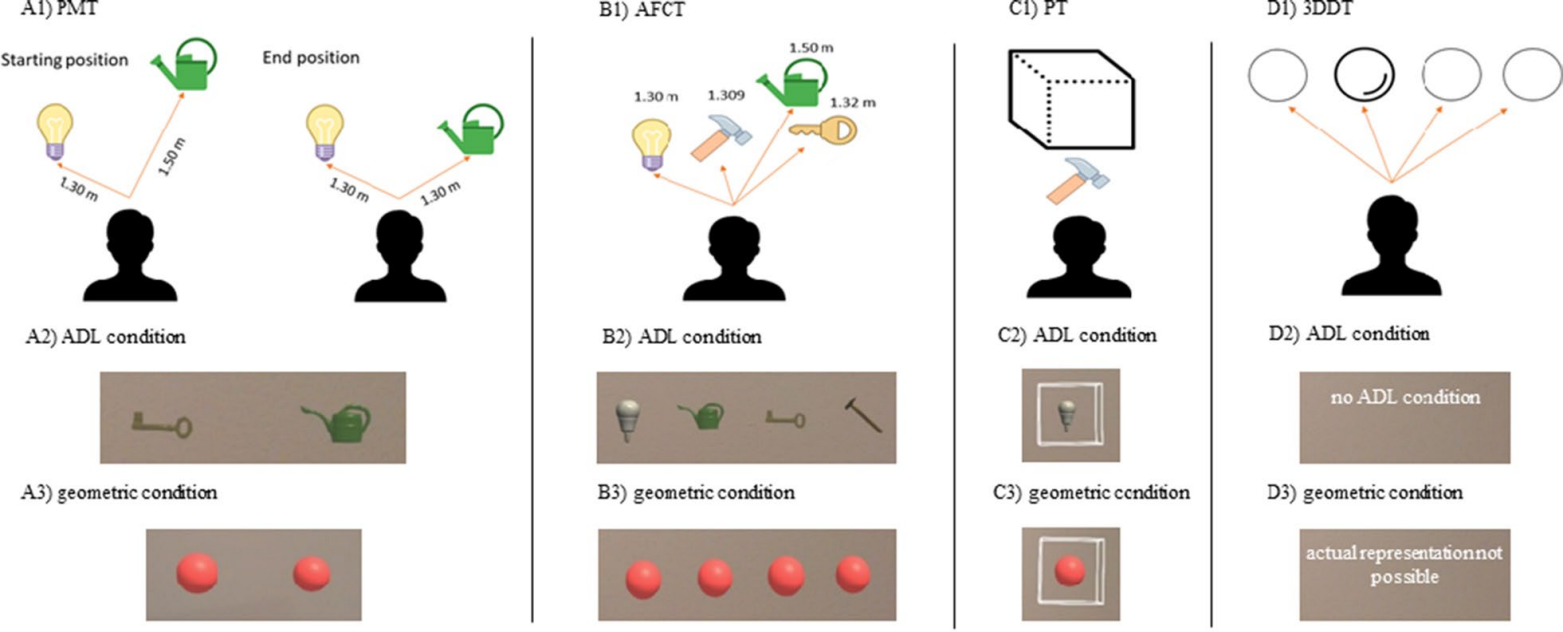
Tasks assessing the ability to judge distance (**A1**–**C1**) and to perceive 3D (**D1**) in AR. For the distance judgment tasks, either objects of Activities of Daily Living (ADL) were used (**A2**–**C2**) or geometric spheres were presented (**A3**–**C3**). **A1**–**D1** show two-dimensional illustrations of the tasks, **A2**–**C2** and **A3**–**C3** show screenshots from the visual field of the user while wearing the Microsoft HoloLens®. CAVE: Screenshots deviate from the real projection of objects via the Microsoft HoloLens®. The impression of depth as displayed with the Microsoft HoloLens® cannot be displayed on a two-dimensional screen

Regarding VSP in an AR environment, two different aspects were assessed: the ability to judge the distances of holographic objects (three tasks: *Perceptual Matching Task (PMT)*, *Alternative Forced Choice Task (AFCT)*, *Position Task (PT)*) and the ability to perceive holographic objects as 3D (one task: *3D Detection Task (3DDT);* Fig. 2). In order for the participants to familiarize themselves with interacting with holographic objects, a familiarization task and two tryouts of each test were performed directly beforehand. Each test was then conducted ten times. Participants answered the tasks verbally. The time needed for task completion was recorded for each test. In the three distance estimation tasks, either geometric holograms (i.e., circle or sphere) or ADL-based holograms (i.e., key, light bulb, watering can and hammer) were presented to the participant with randomized constellation of objects via the Microsoft HoloLens® 1st Generation (Microsoft Corp., Redmond, USA). These ADL objects have been used in previous AR applications for neurorehabilitation within the Therapy Lens project (http://www.therapylens.com/); their programming code is available on GitHub (https://github.com/Ninarohrbach/panto-holo). In the 3D appearance task, solely geometric objects were used (Fig. 2). With the intention to test binocular stereoscopic vison, monocular cues giving information about the distance or 3D appearance of an object were minimized in all four AR tasks.

#### Perceptual matching task

In the PMT, two objects were presented at different distances from the participant. Subjects were instructed to actively adjust the distance of the right object (stimulus object) to the perceived distance of the fixed object on the left (target object) using the scroll wheel of a Bluetooth mouse. As the outcome parameter, the absolute deviation between the stimulus object and the fixed target object was calculated in order to evaluate task performance. The distance between observer and the closest point of each hologram was compared.

#### Alternative forced choice task

During the AFCT, participants were asked to identify which out of four holograms, randomly projected with different distances to the observer, was perceived to be the closest. The difference in distance between the closest and second closest object was between 7 mm and 4 cm, based on the distance detection threshold of healthy subjects in a virtual surrounding [38]. As outcome variable, the proportion of correctly performed test runs was calculated. To evaluate task difficulty, the distance between the closest and the second closest object was given as output for each subtest.

#### Position task

In the PT, the user was asked to name the position of a holographic object that was presented either in front of, in the middle of or behind a translucent cube. The outcome variable was the proportion of correctly performed tasks.

#### 3D detection task

In the 3DDT, four spheres were presented to the user. Three of them were presented at the same depth plane and in two dimensions, while one was projected as popping out, which should be detected by the user. Just as in the *Circles* test, the outcome parameter was the lowest correctly identified stereoacuity level expressed in seconds of arc. The respective stereoacuity levels were then transformed according to the categories of the *Circles* test, ranging from zero to ten.

### Subjective perception of holograms

To assess the participants’ subjective perception of holograms in the AR environment, tested individuals answered the presence questionnaire [39]. Immediately after performing the AR tasks, subjects evaluated the quality of projected holograms by answering the presence questionnaire with respect to objects’ realness and 3D appearance. Furthermore, the questionnaire aims to catch the subjective impression of objects appearing to be present in the real environment. In the SPiAR study, six of Regenbrecht and Schubert’s original seven statements were answered by a seven-point Likert Scale ranging from low presence (0) to high presence (6). One of the original questions addressed the relationship between real and virtual objects. This question was not included in the presence questionnaire of the SPiAR study, as exclusively virtual objects were presented to the participants. Moreover, the second question of the original questionnaire was specified for every hologram (*I had the impression that I could have touched and grasped the hammer/key/light bulb/watering can.*) and added to the questionnaire. The questionnaire was also used to ask for participants’ characteristics, i.e., questions about age, sex, visual impairment indicated by diopter and ocular diseases.

### Data and statistical analysis

The group of patients after stroke and the group of HS were each split into two groups according to their ability to perceive depth, classified by a combination of the results in the *Circles* and *Lang II Stereotest*. The grouping criterion for intact stereovision was a combination of (1) passing the *Lang II Stereotest*, plus (2) reaching at least the 7th cluster of the *Circles* test, which represents 60″. The second group was characterized by impairments in stereovision. Members of this group either failed the *Lang II Stereotest* or were unsuccessful at a stereoacuity level above 60″ in the *Circles* test.

HS as well as patients were described and compared regarding demographic data and VSP. The outcome of the presence questionnaire was used for descriptive statistics in order to describe the sense of presence in the AR environment. Giving an idea of how patients after stroke perceived the holograms, the sense of presence also served as a feasibility indicator of an AR application in this patient group.

With respect to the primary aim of the SPiAR study, outcome variables of each AR task were tested separately for significant differences between subjects with intact stereovision and subjects with impairments in stereovision, as well as between the ADL and the geometric conditions. In case the dependent variable (outcome of AR task) fulfilled the assumptions of normality (Shapiro–Wilk test) or homogeneity of variance (Levene’s test), a mixed-factor ANOVA with one between-group factor (stereovision: stereonormal vs. stereoimpaired) and one within-group factor (task condition: ADL vs. geometric) was calculated. The performance in AR tests, in which one of the assumptions for a mixed-factor ANOVA was violated, was compared between subjects with and without impairments in stereovision using the Mann–Whitney U test. Accordingly, the outcome variable of the AR test was compared between the ADL and geometric conditions by the Wilcoxon test. Differences between stereonormal and stereoimpaired participants in the task completion time were analyzed. As it was not the focus of the study to investigate completion times, participants were not under time pressure, which limits the interpretability of completion times. This data is thus not included in the manuscript, but as a supplementary table.

To address the secondary aim, a linear regression model was applied to predict the performance in those AR tasks where the outcome variable fulfilled the assumption of (1) interval data, (2) linearity, (3) homoscedasticity and no autocorrelation of residuals, and (4) multicollinearity of independent variables. Predictors of this model were the presence or absence of stereovision (combination of *Titmus* and *Lang II Stereotest*), the quality of stereovision in seconds of arc (*Circles* test) and spatial localization/visuoconstruction (VOSP test battery). The relationship between VSP and those AR outcome variables, for which the assumptions for a linear model were violated, but which follow a normal distribution or were on an ordinal scale, were analyzed with the Pearson correlation or Spearman’s rank correlation, respectively.

All data was analyzed using IBM SPSS Statistics version 24 at an α-level of 0.05.

## Results

### Participants’ characteristics

Overall, 20 patients (15 men, 5 women) and 20 HS (10 men, 10 women) participated in the SPiAR trial with a mean age of 64 years (*SD* 14) and 28 years (*SD* 8), respectively. As HS with intact stereovision and HS with impaired stereovision were age-matched, there was no significant difference in age between both subgroups (*z* = − 0.308, *p* = 0.796). Patients with impairments in stereovision (mean age: 70 years, *SD* 7) tended to be significantly older than patients with intact stereovision (mean age: 56 years, *SD* 17; *t* (8.627) =  − 2.163, *p* = 0.060). Of the 20 participating patients, 18 suffered an ischemic stroke and two a hemorrhagic stroke. Eight patients were affected on the right hemisphere, four patients on the left, four patients on both hemispheres and four patients on the brainstem. Different spatial and visual/ocular-motor impairments were diagnosed: neglect (n = 2) hemianopia (left (n = 1), left and right (n = 1)), near exophoria (n = 1), heterotropia (n = 1), nystagmus left (n = 1) and reduced visual acuity (n = 2). Additional detailed patient-specific information and quantification of the visual impairments is provided in Table 1.

**Table 1:** Patients' characteristics

ID	Sex	Age [years]	Stereovision	VOSP	Type	Location of infarction	Visual impairment	Patients’ comments
P01	f	66	Impaired	50	I	Right capsula interna (next to thalamus)	–	–
P03	m	60	Impaired	47	I	Left brainstem	Saccadic pursuit, gaze evoked nystagmus to the left, near exophoria with double images, hemianopia left	PMT: difficult to match hammer and watering can
P04	f	66	Impaired	50	I	Left middle and anterior cerebral artery	–	–
P06	m	67	Impaired	45	I	Right middle cerebral artery, V4, MT/V5, intraparietal area, inferiortemporal area	Neglect, hemianopia	–
P07	m	77	Intact	48	I	Multiple focal infarction	–	Size of ADL objects was irritating
P08	m	42	Intact	40	I	Thalamus, both sides (+ cerebellar lesion right)	–	Difficulties in ADL condition
P09	m	53	Intact	45	I	Right middle cerebral artery	–	–
P11	f	83	Impaired	46	I	Pons, left	–	–
P13	m	52	Intact	48	I	Right frontal, parietal area both sides, V1, V2, V4, MT/V5, intraparietal area	–	Felt discomfort while wearing Microsoft HoloLens®, heavy
P15	m	36	Intact	42	I	Left frontoparietal, left middle cerebral artery, MT/V5, intraparietal area, inferiortemporal area	–	–
P16	m	69	Impaired	37	I	Right middle cerebral artery	Uncorrected reduction in visual acuity on both eyes (greater in near distances (30%) than far distances (50%))	–
P17	m	82	Impaired	36	I	Left middle cerebral artery, inferiortemporal area	–	–
P18	m	47	Intact	43	I	Right frontal and occipital area, V2, V4, MT/V5	–	–
P19	m	70	Impaired	47	I	Mesencephalon and pons left	–	–
P20	f	64	Impaired	50	I	Medulla oblongata, right	Heterotropia, prism correction glasses (8 dpt) for left eye reduce visual acuity during near distance testing	Enjoyed testing
P21	m	56	Intact	49	I	Nucleus caudatus, capsula interna left (dorsal), LGN	–	–
P23	f	86	Intact	45	H	Basal ganglia, left + Thalamus, both sides, LGN	–	–
P24	m	65	Impaired	50	H	Basal ganglia, right, LGN	–	Was interested
P25	m	76	Impaired	45	I	Medulla oblongata, right	Concentric narrowing of the visual field	–
P27	m	71	Impaired	37	I	Right middle cerebral artery, MT/V5, intraparietal area, inferiortemporal area	Neglect	Cannot see all four points during *Titmus-Test*

*ADL* Activities of Daily Living, *AR* Augmented Reality, *f* female, *H* hemorrhagic; *I* ischemic, *m* male, *PMT* *Perceptual Matching Task*, *VOSP* Visual Object and Space Perception, *y* years

Around half of the study population (50% of HS, 40% of patients) passed both the *Lang II* and *Circles* test and were classified to have full stereovision. As a measurement of the quality of stereovision, patients achieved a mean category of 6 (≙ 100″; *SD* 3) in the *Circles* test and HS a mean category of 7 (≙ 80″; *SD* 3). Ceiling effects were found for spatial localization/visuoconstruction in HS, who reached a median score of 50 points (*IQR* 49–50) in the VOSP test battery. Stereovision did not affect spatial perception of HS, as there was no significant difference in the VOSP result between HS with intact and impaired stereovision (*z*_*VOSPsum*_ =  − 1.088, *p* = 0.353; *z*_*Cube Analysis*_ =  − 1.453, *p* = 0.481; *z*_*Dot Counting*_ =  − 0.610, *p* = 0.739; *z*_*Number Location*_ =  − 1.641, *p* = 0.218; *z*_*Position Discrimination*_ =  − 1.000, *p* = 0.739). Similarly, there was no significant difference in spatial localization/visuoconstruction between patients with intact stereovision and patients with stereoimpairments (*z*_*VOSPsum*_ =  − 0.545, *p* = 0.624; *z*_*Cube Analysis*_ =  − 0.359, *p* = 0.734; *z*_*Dot Counting*_ < 0.001, *p* = 1.000; *z*_*Number Location*_ =  − 1.544, *p* = 0.135; *z*_*Position Discrimination*_ =  − 0.509, *p* = 0.678). Overall, patients received a median of 46 points (*IQR* 43–49) in the VOSP.

The reported sense of presence in the AR environment indicated that both groups, HS and patients, perceived virtual objects as touchable and graspable (*med:* 5; *IQR* 2–5). While the study population was undecided about the possibility to interact with the hammer (*med*: 3; *IQR* 1–5) or the key (*med*: 3; *IQR* 2–5), a rather high presence was reported when the watering can (*med:* 4; *IQR* 2–6) or the light bulb (*med:* 4; *IQR* 2–6) was presented. Participants had the impression that virtual objects in the AR environment were located in space (*med:* 5; *IQR* 3–5) and they perceived the holograms as 3D (*med*: 5; *IQR* 4–6). Neither patients nor HS paid attention to differences between reality and virtuality (*med*: 4; *IQR* 1–6) nor did they need much effort to recognize the holograms as 3D (*med*: 5; *IQR* 2–6). Solely, the judgment about the perceived realness of holographic objects was different between HS and patients. With a median of 4 (*IQR* 2–5), HS judged the realness of holograms lower than patients did (*med:* 6, *IQR* 5–6).

### Effects of stereovision on the perception of holograms

#### Distance estimation

##### Perceptual Matching Task

**Fig. 3:**
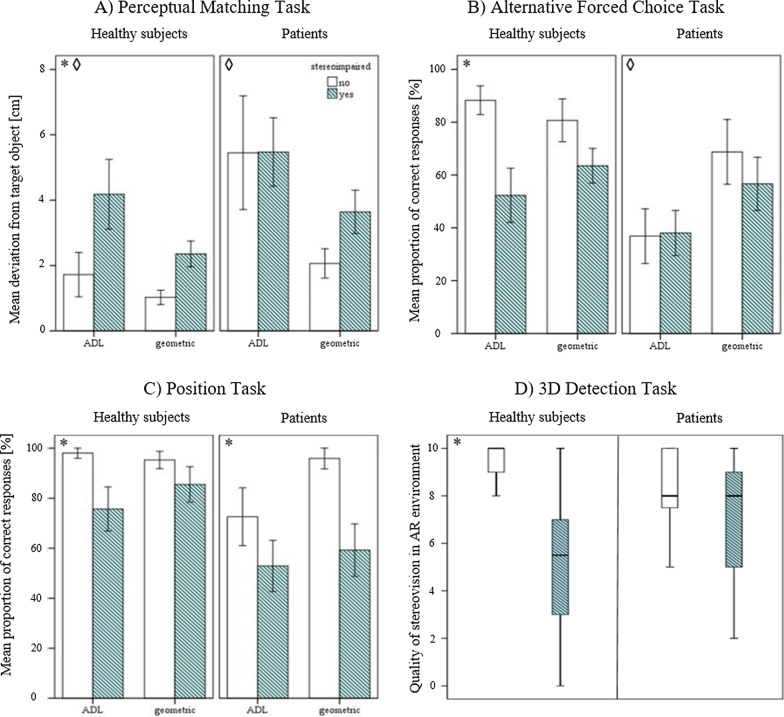
Results of the tests in the AR environment. The performance is presented separately for healthy subjects and patients. Error bars in **A**–**C** represent ± one standard error. *Significant difference in task performance between stereointact and stereoimpaired participants. ◊ Significant difference in task performance between the ADL and the geometric conditions

The deviation of the stimulus object from the fixed target object in the end position was an indicator for the subjects’ performance in the PMT. HS had a mean deviation from the target object of 2.31 cm (*SD* 1.87). The average deviation in the PMT of HS with impaired stereovision was significantly higher than the average deviation of HS with intact stereovision (*F*Stereovision (1, 18) = 6.351, *p* = 0.021, *η*^2^ = 0.021). Besides stereovision, the task condition had a large significant effect on task performance of HS (*F*Condition (1, 18) = 4.674, *p* = 0.044, *η*^2^ = 0.206). As visualized in Fig. 3A, the mean deviation of the stimulus object from the target object in the end position was smaller in the geometric condition, while HS had more difficulties in matching ADL objects. There was no significant interaction between the factors task performance and stereovision (*F*Condition*Stereovision (1, 18) = 0.933, *p* = 0.347, *η*^2^ = 0.049).

Patients aligned the stimulus object, on average, with a mean deviation of 4.40 cm (*SD* 2.84) from the target object. Within the patient group, presence or absence of stereovision had no significant effect on the mean deviation from the target object (*F*Stereovision (1, 18) = 0.394, *p* = 0.538, *η*^2^ = 0.021). In contrast, task condition had a large significant effect on the matching accuracy in the PMT (*F*Condition (1, 18) = 11.586, *p* = 0.003, *η*^2^ = 0.392). Similar to HS, patients matched ADL objects with a higher mean deviation from the target object than geometric objects (Fig. 3A). Regarding the performance of patients in the PMT, the factors task condition and stereovision showed no significant interaction (*F*Condition*Stereovision (1, 18) = 1.028, *p* = 0.324, *η*^2^ = 0.054).

When analyzing the relationship between the ADL and geometric condition of the PMT in the subgroup of participants with stereoimpairments, no significant correlation was present in HS (*r* (10) = 0.496, *p* = 0.145). HS who aligned both objects with less deviation in the geometric condition did not necessarily perform well in the ADL condition. In the group of patients with stereoimpairments, results revealed a strong, significant correlation between the ADL and geometric condition (*r* (12) = 0.578, *p* = 0.049). Patients with problems in the ADL condition of the PMT also showed deficits in aligning geometric objects.

##### Alternative Forced Choice Task

For the evaluation of the AFCT, the proportion of correctly answered tasks was analyzed to evaluate subjects’ performance. On average, HS answered 70% (*SD* 22%) of AFCTs correctly. In HS, stereovision had a large significant effect on the proportion of correct responses in the AFCT (*F*Stereovision (1, 18) = 13.738, *p* = 0.002, *η*^2^ = 0.433). HS with intact stereovision were, with a mean proportion of 84% (*SD* 15%), more often successful in detecting the closest hologram compared to stereoimpaired HS with a mean proportion of 56% (*SD* 18%; Fig. 3B). Unlike stereovision, the task condition did not significantly affect the performance in the AFCT in HS (*F*Condition (1, 18) = 0.046, *p* = 0.833, *η*^2^ = 0.003). The interaction between the factors stereovision and task condition was not significant (*F*Condition*Stereovision (1, 18) = 1.248, *p* = 0.279, *η*^2^ = 0.065).

On average, patients answered 49% (*SD* 25%) of AFCTs correctly. Those patients with intact stereovision performed the AFCT with a mean proportion of 54% (*SD* 30%) similar to patients with impaired stereovision, who identified the closest object in 47% (*SD* 23%) of tasks. Thus, stereovision had no significant effect on the task performance in patients after stroke (*F*Stereovision (1, 18) = 0.193, *p* = 0.666, *η*^2^ = 0.011). As visualized in Fig. 3B, patients showed difficulties especially in the ADL condition of the AFCT, while a relatively high proportion of tasks was correctly answered in the geometric condition. The task condition had a large significant effect on the performance of the AFCT in the patient group (*F*Condition (1, 18) = 10.087, *p* = 0.007, *η*^2^ = 0.183). Similar to the HS group, there was no significant interaction between task condition and stereovision (*F*Condition*Stereovision (1, 18) = 0.697, *p* = 0.415, *η*^2^ = 0.037).

Notably, there was no significant relationship between the difficulty of the AFCT (indicated by the difference in distance between the closest and second closest object) and the proportion of correct responses (*rs* (40) = − 0.061, *p* = 0.707), neither in the ADL condition (*rs* (40) = − 0.012, *p* = 0.939) nor in the geometric condition (*rs* (40) = − 0.210, *p* = 0.193). Additionally, the difficulty in the AFCT did neither significantly differ between the group of stereointact HS and stereoimpaired HS (*z* = − 0.302, *p* = 0.762) nor between stereointact patients and stereoimpaired patients (*z* = − 0.906, *p* = 0.365).

When analyzing the relationship between task performance in the ADL and geometric condition of the AFCT, results revealed no significant correlations, neither in HS (*r* (10) = − 0.256, *p* = 0.476) nor in patients (*r* (12) = 0.392, *p* = 0.208).

##### Position task

For the evaluation of the PT, the proportion of correctly named positions of the object in relation to the cube was analyzed to evaluate task performance. HS correctly answered, on average, a proportion of 89% (*SD* 16%) of tasks. Stereovision significantly affected the performance of HS in the PT. Stereointact HS succeeded, with a mean proportion of 97% (*SD* 5%), significantly more often than stereoimpaired HS with a mean proportion of 81% (*SD *20%; *z* = − 1.994, *p* = 0.046). Stereonormal HS performed significantly better than HS with impairments in stereovision, especially in the ADL condition (*z* = − 2.352, *p* = 0.019; Fig. 3C). However, the overall performance in the *Position Task* did not significantly differ between the ADL and geometric conditions in HS (*z* = − 0.280, *p* = 0.779).

Patients succeeded, on average, in 66% (*SD* 30%) of PTs. As visualized in Fig. 3C, patients with intact stereovision performed significantly better in the PT than stereoimpaired patients (*z* = − 2.031, *p* = 0.042). Unlike HS, this effect of stereovision was mainly present in the geometric condition of the PT (*z* = − 2.602, *p* = 0.009). Within patients, the difference between ADL and geometric conditions tended towards significance (*z* = − 1.726, *p* = 0.084). With a mean proportion of 74% (*SD* 34%), the position of geometric objects was more often detected than the position of an ADL object with a mean proportion of 61% (*SD* 35%).

The positioning of ADL or geometric objects is an indicator for the task difficulty level (higher difficulty level for positioning of objects behind the cube). Objects were randomly placed in front of, in the middle of or behind the translucent cube. Positioning of objects in relation to the cube was similarly distributed in the group of HS with and without impairments in stereovision as well as in stereonormal and stereoimpaired patients after stroke. Concretely, on the group level the stimulus object was positioned in front of the cube as frequently as it was projected in the middle of or behind the cube. On the individual level an disproportional high number of tasks in which the stimulus object was located behind the cube was presented to three healthy participants with impairments in stereovision, one healthy individual with intact stereovision, and four patients with stereoimpairments. However, there was no significant difference in the proportion of correctly answered tasks between subtasks where the sphere or the ADL object was positioned in front of the cube, in the middle of the cube or behind the cube, neither within stereonormal HS (*F* (2, 117) = 0.601, *p* = 0.550, *η*^2^ = 0.010) or stereoimpaired HS (*F* (2, 117) = 0.648, *p* = 0.525, *η*^2^ = 0.011) nor in stereonormal patients (*F* (2, 93) = 0.564, *p* = 0.571, *η*^2^ = 0.012) or stereoimpaired patients (*F *(2, 141) = 0.434, *p* = 0.649, *η*^2^ = 0.006).

When analyzing the relationship between the performance in the ADL and geometric conditions of the PT, results reveal no significant correlations, neither within the group of HS (*r* (10) = 0.186, *p* = 0.607) nor in the patient group (*r* (12) = 0.479, *p* = 0.115).

#### 3D appearance

For the evaluation of the 3DDT, the identified stereoacuity level was analyzed with values ranging from 0 (≙ no stereovision) to 10 (≙ 40″). HS showed a median quality of stereovision of 9 (*IQR* 6–10). As shown in Fig. 3D, stereointact HS reached a median value of 10 (*IQR* 9–10), which is a significantly higher stereoacuity level than HS with stereoanomalies, who reached a median stereoacuity level of 6 (*IQR* 3–7; *z* = − 2.936, *p* = 0.003).

Patients reached a median quality of stereovision of 8 (*IQR* 6–10) in the 3DDT. Quality of stereovision in the AR environment did not differ significantly between patients with and without stereoimpairments (*z* = − 1.000, *p* = 0.317). With a median stereoacuity of 8 (*IQR* 5–9), stereoimpaired patients performed comparable to stereonormal patients with a median value of 8 (*IQR* 8–10).

### Predictors of visuospatial perception in the Augmented Reality environment

#### Distance estimation

The performance of HS in the first distance estimation task, the PMT, was significantly predicted by a linear regression model with the predictors stereovision, quality of stereovision and spatial localization/visuoconstruction (*F* (3, 19) = 12.541, *p* < 0.001). The combination of the three predictors explained 70.2% of variance in the deviation from the stimulus to the target object. Exclusively, the quality of stereovision turned out to be a highly significant predictor of the deviation from the target object (*p* < 0.001; Table 2). In the ADL condition (*F* (3, 19) = 7.602, *p* = 0.002) as well as in the geometric condition (*F* (3, 19) = 4.718, *p* = 0.015), the linear model significantly predicted the performance of HS in the PMT. The model explained 58.8% of variance in the deviation from the target ADL object. The quality of stereovision of HS predicted their performance in the ADL condition of the PMT (*p* = 0.002; Table 2). When matching the distance of two geometric objects, the model explained 46.9% of variance in task performance. In the geometric condition, there was the trend that quality of stereovision significantly predicted the deviation from the target object (*p* = 0.058; Table 2).

**Table 2:** Predictors of task performance in the AR environment

	Patients			Healthy subjects		
	PMT	AFCT	PT	PMT	AFCT	PT
Total						
Stereovision	(0.116)	(− 0.616)	− 0.236	− 0.463	− 0.092	0.139
Quality of stereovision	(− 0.023)	(− 0.527)	0.268	− 1.189**	0.709*	0.879**
Spatial localization/visuoconstruction	(− 0.407)	(0.246)	0.423	− 0.041	− 0.085	− 0.277
ADL						
Stereovision	(− 0.073)	(− 0.217)	(0.091)	− 0.561	0.135	0.392
Quality of stereovision	(− 0.084)	(− 0.263)	(0.414)	− 1.173**	0.770*	1.150**
Spatial localization/visuoconstruction	(− 0.252)	(− 0.323)	(0.118)	0.028	0.231	− 0.218
Geometric						
Stereovision	0.002	(− 0.955)	− 0.450	− 0.117	(− 0.277)	(− 0.463)
Quality of stereovision	− 0.401	(− 0.862)	0.101	− 0.731	(0.193)	(− 0.108)
Spatial localization/visuoconstruction	− 0.508*	(0.271)	0.533**	− 0.218	(− 0.232)	(− 0.271)

Cells present *β*-coefficients of each predictor.*significant predictor at *p* < 0.05, **significant predictor at *p* < 0.01, (): model did not significantly predict task performance, ADL: Activities of Daily Living, AFCT: *Alternative Forced Choice Task*, PMT: *Perceptual Matching Task,* PT: *Position Task,* Spatial localization/Visuoconstruction: VOSP test battery, Quality of stereovision: *Circles* test, Stereovision: stereoimpaired stereointact

Within the patient group, neither the model for the PMT in general (*F* (3, 19) = 1.240, *p* = 0.328) nor the model for the ADL condition of the PMT was significant (*F* (3, 19) = 0.418, *p* = 0.742). The deviation from the target object in the PMT was only predictable by the linear model in the geometric condition (*F* (3, 19) = 5.537, *p* = 0.008). Here, 50.9% of variance in patients’ performance could be explained by the predictors stereovision, quality of stereovision and spatial localization/visuoconstruction. A noteworthy result is that only the latter predictor was significant for the deviation from the target sphere (*p* = 0.018; Table 2). Specifically, two subtests of the VOSP test battery were significantly associated with the matching accuracy in the geometric condition of the PMT, namely Number Location (*r*s (20) = − 0.512, *p* = 0.021) and Cube Analysis (*r*s (20) = − 0.473, *p* = 0.035).

Within the group of HS, the performance in the AFCT was predictable by the linear model (*F* (3, 19) = 8.357, *p* = 0.001). VSP explained 61.0% of variance in the proportion of correct responses. While participants’ stereovision (*p* = 0.780) as well as their result in the VOSP test battery (*p* = 0.635) could not be used to significantly prognosticate the performance in the AFCT, quality of stereovision was a significant predictor (*p* = 0.034; Table 2). When ADL objects were presented to HS, 50.7% of variance in the proportion of correct responses was explained by the linear model (*F* (3, 19) = 5.476; *p* = 0.009). Again, quality of stereovision was the only significant predictor for the proportion of correctly identified ADL objects (*p* = 0.040; Table 2). On the contrary, the linear model was not able to significantly predict the performance of HS in the geometric condition of the AFCT (*F* (3, 19) = 1.373, *p* = 0.287). The linear model failed to predict the performance of patients in the AFCT (*F* (3, 19) = 0.493, *p* = 0.692). The three predictors did not apply for modeling the task performance, neither when ADL (*F* (3, 19) = 0.506; *p* = 0.683) nor when geometric objects were presented to the patient (*F* (3, 19) = 0.996, *p* = 0.420).

The prediction of the performance of HS in the PT was significant (*F* (3, 19) = 9.297, *p* < 0.001). Sixty-three percent of variance in the proportion of correct responses were explained by the three predictors stereovision (*p* = 0.633), quality of stereovision (*p* = 0.009) and spatial localization/visuoconstruction (*p* = 0.124; Table 2). While the model did not apply for the geometric condition of the PT (*F* (3, 19) = 0.852, *p* = 0.486), the three predictors were highly significant in prognosticating the performance of HS when ADL objects were presented (*F* (3, 19) = 17.776, *p* < 0.001). In the latter condition, the linear model explained 76.9% of variance in the proportion of correctly identified positions of ADL objects in relation to a translucent cube. Again, quality of stereovision exclusively predicted the performance in the ADL condition of the PT (*p* < 0.001), while stereovision and the result of the VOSP test battery could not be used to prognosticate task performance in the geometric condition (Table 2).

Regarding the performance of patients in the PT, the linear model significantly explained 46% of variance in the proportion of correct responses (*F *(3, 19) = 4.565, *p* = 0.017). When analyzing the three predictors separately, results revealed that only spatial localization/visuoconstruction tended to significantly predict the performance in the PT (*p* = 0.053; Table 2). While the linear model did not significantly predict the performance of patients in the ADL condition of the PT (*F* (3, 19) = 0.895, *p* = 0.465), it explained 59.9% of variance in the proportion of correct responses in the geometric condition (*F* (3, 19) = 7.959, *p* = 0.002). A noteworthy result was that the VOSP test battery was a highly significant predictor for the proportion of correctly located spheres in relation to the translucent cube (*p* = 0.008; Table 2), while none of the four subtasks (i.e. Dot Counting, Position Discrimination, Number Location, Cube Analysis) were related to patients’ performance in the geometric condition of the PT.

#### 3D appearance

Spatial localization/visuoconstruction was not related to the performance in the 3DDT, neither in the group of HS (*r*s (20) = − 0.180, *p* = *0.4*48), nor in the patient group (*r*s (20) = 0.059, *p* = 0.804). In contrast, the quality of stereovision of HS with stereoimpairments was highly related to the perception of holographic objects as 3D (*r*s (10) = 0.876, *p* = 0.001). The higher the quality of stereovision of the healthy participant, the better the performance in the 3DDT. Quality of stereovision of patients after stroke with stereoimpairments tended to be significantly associated with the ability to perceive holograms as 3D in the 3DDT (*r*s (12) = 0.569, *p* = 0.054).

## Discussion

In summary, it is not only the presence of stereovision but also the quality of stereovision of HS that has an influence on the ability to judge the distance of holograms and their perception as 3D, while spatial localization/visuoconstruction had no impact on task performance. Based on this finding, the validity of the developed AR tasks in assessing stereovision is assumed. In patients after stroke, impaired stereovision affected the performance in one of three distance estimation tasks. While the quality of stereovision did not significantly predict the ability to accurately judge distances in the AR environment, spatial localization/visuoconstruction significantly predicted the performance in the geometric condition of distance estimation tests. The patients’ ability to perceive holograms as 3D was neither affected by nor related to any aspect of VSP.

### Visuospatial perception and presence in context of the state of research

Stereovision was analyzed as one aspect of VSP. Recruitment of healthy individuals focused on matching the proportion of subjects with intact and impaired stereovision. Therefore, in the group of HS, impairments in stereovision were present in 50% of participants and the mean stereoacuity level was 80″. With respect to norm values, HS showed a lower quality of stereovision than healthy adults in a similar age range, who had a mean stereoacuity level around 43″ in the *Circles* test [40, 41]. With a mean stereoacuity level of 100″, patients who participated in the SPiAR trial are comparable to previous data from a similarly healthy age group with a mean stereoacuity level of 94″ [42]. However, the patient group shows higher variation in the stereoacuity level (*SD* 400'') than healthy individuals from the same age group (*SD* 60''). Nevertheless, the patient group in the SPiAR study performed slightly better in the *Circles* test than a population of patients after stroke with reduced convergent fusion who had a mean stereoacuity level of 147″ (*SD* 68″ (4)). One interesting observation during the recruitment of participants was that it took effort to recruit HS with reduced stereovision to achieve a 50/50 ratio of stereointact and stereoimpaired subjects. On the contrary, during the recruitment of patients after stroke, it was harder to find patients with intact stereovision, as the majority showed stereoimpairments. This finding is consistent with the state of research, indicating a higher prevalence for complete stereoblindness in stroke survivors (21% (16)) than in HS (7% (32)).

As the second aspect of VSP, spatial localization/visuoconstruction was assessed by through the VOSP test battery. HS showed a ceiling effect in the performance of all four tasks, which is supported by norm data [36]. Patients showed impairments in the Dot Counting, the Position Discrimination task and the Cube Analysis, which is in accordance with previous data [43]. Patients’ performance in the Number Location task varied greatly, ranging from 3 to 10 points. According to existing literature, patients with right hemispheric brain damages show deficits in the Number Location task, while the performance of patients with left hemispheric brain damages does not differ from HS [44].

Results of the presence questionnaire showed that the study population had a low sense of presence specifically when either the hammer, the key or the light bulb were presented. This is most likely due to the projection of ADL objects without their natural proportions. Overall, HS perceived the objects as located in space. Neither patients nor HS were strained when perceiving the holograms as 3D and did not pay attention to differences between reality and virtuality. In summary, patients and HS had an overall high sense of presence in the AR environment indicating that both groups perceived the holograms as intended. Feasibility of the AR tasks with respect to accurate perception of holograms can be assumed for patients and HS of the SPiAR study.

### The impact of visuospatial perception on distance estimation and 3D appearance of holograms

The perception of virtual holograms was associated with VSP of healthy participants as well as patients after stroke.

#### Healthy subjects

In the population of HS, impaired stereovision had an effect on the ability to estimate the distance of holograms. Concretely, the matching accuracy in the PMT, the detection of the closest object as well as the detection of the position of a hologram were all performed better with intact stereovision. These findings validate the test’s ability to assess a persons’ capability in stereoscopic distance judgment in an AR environment, since HS with stereoimpairments perceived the distance of holograms less accurately than HS with intact stereovision.

It is interesting to note that HS showed better matching accuracy when geometric objects were presented compared to ADL objects. In the ADL condition, objects were presented to the participant without scaling the objects’ size according to their natural appearance. Therefore, true stereoscopic vision was necessary to solve this task and to match the depth planes of both objects. In contrast, two identical spheres were projected in the geometric condition. Here, the size of the spheres changed according to the distance from the user, which might have facilitated the subject to estimate changes in distance. However, the effect of impaired stereovision appeared independent of the presented object type in each of the three distance estimation tasks.

VSP of HS significantly predicted the performance in all three distance estimation tasks and in their ADL condition. Here, quality of stereovision turned out to be a significant predictor of task performance. In contrast, spatial localization/visuoconstruction did not predict the result in the AR test which can be ascribed to the ceiling effect in the VOSP test battery.

Regarding the perception of holograms as 3D, impaired stereovision showed an effect in the group of HS. The quality of stereovision in the AR environment was higher in participants with intact stereovision compared to those with stereoimpairments. Additionally, the subject’s quality of stereovision was highly associated with the performance in the 3DDT. Conclusively, the SPiAR trial confirms the previous statement that HS with intact stereovision are able to accurately perceive depth when wearing the Microsoft HoloLens® [27]. Although the *Circles* test, which assesses quality of stereovision, was performed at a distance of 40 cm, while the spheres in the 3DDT were projected at a distance of 1.5 m, the measured stereoacuity level in the *Circles* test was highly related to the 3DDT. Results of a previous study, in which the performance in a stereotest was compared between near and far distance, also revealed a high correlation of the test results in HS [45]. In contrast, spatial localization/visuoconstruction was not related to the perception of holograms as 3D, due to the ceiling effect in HS.

#### Patients

In patients after stroke, impaired stereovision had a significant effect on the ability to judge the distance of a holographic object in the PT. In our investigated patient group, the effect of impaired stereovision was particularly present in the geometric condition of the PT, whereas the performance in the ADL condition was not sensitive to affected versus unaffected stereovision. Hence, it can be hypothesized that there is another cognitive aspect involved when the distance of ADL objects has to be estimated. On the basis of a dual-stream visual processing (ventral and dorsal), different cortical areas might get activated when estimating the distance of ADL objects. In contrast to the geometric condition, object use and interpretation of objects might be subconsciously processed during the ADL condition, which involves an additional layer of visual processing [12]. Under the involvement of the dorsal stream, the distance estimation of ADL objects might not be independent of size interpretation and thus be influenced by the unnaturally scaling of objects’ size. This hypothesis is emphasized by the patients’ response to the presence questionnaire and their comments during the testing procedure: Patients after stroke did not perceive holographic objects as natural and some mentioned that it was difficult for them to suppress the intention of naming the biggest object instead of the closest. Assuming the above-mentioned dual-stream visual processing in the ADL condition, findings from brain imaging data support the hypothesis of biased distance estimation due to unconscious size interpretation of objects. Some patients showed damages in the inferiortemporal area of the ventral stream (see Table 1) which is responsible for size constancy [8]. The inclusion of patients with and without damages in this brain area might be a factor that influenced distance estimation of ADL objects.

The performance in the PMT and the AFCT was neither affected by stereovision nor predicted by the quality of stereovision. The reason why impaired stereovision had no effect on distance judgments in the PMT and AFCT in the patient group most likely lies in the patients’ characteristics (see Table 1). In four patients, the assessment of stereoscopic vision was influenced by near exophoria with double images, uncorrected reduction in visual acuity, prism correction glasses which cause a difference in visual acuity between the left and the right eye and hemianopia. These visual/ocular-motor impairments make it difficult to compare and relate the results of near and far stereovision tests. The patient with near exophoria perceives double images in near distances, which negatively affects the performance in the clinical stereotests (at a distance of 40 cm), while AR testing (at a distance of 1.5 m) remains unaffected. Further, the testing of one patient was impaired by uncorrected reduction in visual acuity, which was less severe for distant vision in the AR tests compared to close distances in the clinical stereotests. Since visual acuity influences the quality of stereovision [45], the patient’s uncorrected vision biased the effect of stereovision on distance estimation in the AR environment. During clinical stereotesting, one patient wore glasses with prism correction for the left eye, which reduces visual acuity. The resulting difference in visual acuity between both eyes is known to negatively affect stereovision [9]. The diagnosis of hemianopia in another patient also makes it difficult to compare the results of the clinical stereotests with AR tasks. Visual field deficits, resulting from hemianopia, potentially hinder the binocular fusion of the smaller circles in the *Titmus-Test*, while the spheres in the AR tests were bigger in size and thus provide a higher chance to be at least partly fused. Reasonably, the relationship between stereoimpairments (tested at a distance of 40 cm) and distance judgment in the AR environment (assessed at a distance of 1.5 m) is biased by the visual/ocular-motor impairments of those four patients.

Independent of stereovision, patients after stroke showed better distance estimation when geometric objects were presented compared to ADL objects. The matching accuracy in the geometric condition of the PMT significantly correlated with the performance in the ADL condition, i.e., patients with less accurate distance estimation in the ADL condition also performed worse in the geometric condition. Thus, impairments in VSP were even more evident when ADL objects were presented. This finding is relevant for therapy, in which ADL tasks are frequently practiced [24, 26].

Results of the VOSP test battery significantly predicted the performance in the geometric condition of two distance estimation tasks: the PMT and the PT. Spatial localization/visuoconstruction significantly predicted the ability to judge distances in those AR tasks. However, VSP did not predict the ability to estimate distances in the AFCT. Patients after stroke showed difficulties in detecting the closest object in the AFCT, independent of visuospatial abilities. The task was potentially too difficult as the minimum difference between the closest and second closest object was defined based on data from healthy subjects. The distance detection threshold in a virtual surrounding was found to be 7 mm [38]. However, whether this threshold is appropriate for patients after stroke was not investigated. Nevertheless, there was no relationship between task difficulty (difference between closest and second closest object) and the proportion of correct responses in the AFCT on the group level. Still, results are potentially biased as it is possible that some individuals randomly received a disproportional high amount of AFCTs with maximal difficulty (7 mm between closest and second closest object).

Patients’ ability to perceive objects as 3D was neither associated with nor affected by their VSP. The performance in the 3DDT was not related to visuoconstruction/spatial localization skills. Neither stereovision nor the quality of stereovision affected the detected stereoacuity level in the 3DDT. These findings can again be explained by patients’ visual/ocular-motor impairments, which have a different impact on stereovision in close distances compared with further distances.

## Strengths and limitations

There are some limitations in the methodology of the SPiAR study, which impede the interpretability of the results. The group of HS was not planned to be comparable in age to the group of patients. The group of HS served exclusively as a feasibility indicator and confirmed the validity of the AR tasks with respect to the assessment of stereoscopic vision. For an unbiased comparison of VSP in the AR environment between stereoimpaired and stereointact HS, it was advantageous that these two groups were matched regarding age and sex.

Presumably, there were some factors influencing the performance in AR tasks: the use of differently shaped ADL objects in the PMT, the difficulty in the AFCT and the positioning of the stimulus object in the PT. In the PMT, the projected ADL objects had different shapes and therefore varied in thickness. In contrast to the hammer and the key, the light bulb and the watering can were characterized by their round body and were consequently thicker. One could criticize that matching a comparably thin object with a thicker one is more difficult than aligning the distance of two holograms with greater similarity. However, it has been shown that the matching accuracy of HS in an AR environment is independent of the stimuli shape [38]. In the AFCT, not every participant received the same task difficulty on average. Fortunately, the mean difficulty of the AFCT was not related to task performance on the group level. However, on the individual level, there was the potential for accumulation of very difficult tasks for some participants. Random positioning of the stimulus object in the PT influenced the proportion of tasks where uncrossed disparities had to be detected (object behind the cube). Subjects with intact stereovision might show problems in detecting the direction of specifically uncrossed disparities [46]. Nevertheless, the frequency of tasks where the stimulus object was positioned in front of, in the middle of or behind the translucent cube did not differ between HS and patients with intact or impaired stereovision. On the individual level, the number of tasks where uncrossed disparities were presented was disproportionally high for some participants, which might have influenced the results.

There is evidence to suggest that the absence of a visual acuity assessment of all participants was a limitation of the study. Perhaps, there was an influence of minor deficits in visual acuity on the stereoacuity level, especially on distant stereoacuity testing in the AR environment [45]. It can be assumed that there were at least no gross deficits in visual acuity in the SPiAR trial, as the researcher checked whether the participants were able to read task instructions and asked whether the objects can be seen sharply.

It is plausible that the researcher’s inability to see what was projected on the visual field of the participant by the semi-silvered mirrors of the Microsoft HoloLens® limited the feasibility of AR tests. A duplication of the AR projections on a separate desktop screen would have been beneficial for the researcher in order to check whether the Microsoft HoloLens® was positioned appropriately and in order to check if technical problems occurred. Further, the opportunity to observe what the user saw in real-time would have reduced the need to rely on the participant’s verbal feedback. Technical failure or misunderstanding of the task on the part of the participant could have been detected by the researcher without communication. Since the visual field of the SPiAR study patients was not duplicated on a second screen, the inclusion of patients with severe aphasia was hindered.

Nevertheless, the high feasibility of the SPiAR study in testing stereopsis using a modern technology in an elderly study population is highly valued. Since the older generation is generally characterized as being afraid of, and feeling insecure and incompetent with the use of technology [47, 48], it was all the more gratifying that patients after stroke were interested in the chosen device and enjoyed the testing. The acceptability of such a modern technology in testing stereoscopic vision in elderly patients was high. The use of a Bluetooth mouse turned out to be a feasible choice, as it provided an easy way to interact with the holograms. Additionally, there was no severe technical failure, which is important to increase the patients’ motivation and to avoid patients’ frustration due to a malfunctioning system [49].

Another strength of the SPiAR study is the design used to assess true stereoscopic vision in an AR environment where the presence of monocular depth cues, which enables one to interpret depth by the input of one eye, was reduced. One example of this is the reduction of the monocular depth cue size. In the ADL condition, objects had different shapes and their relative size was not scaled according to their natural appearance. Thus, the monocular cue of objects’ size did not provide any information about the distance of ADL objects. In the geometric condition, identical spheres were projected. One could hypothesize that the size of the geometric objects served as monocular depth cue. However, the difference in distance of objects from the observer (which was 4 cm or less) was below the threshold that could be detected by the interpretation of the objects’ size as a monocular depth cue. According to Cutting et al., relative size of similar objects facilitates the discrimination of distances between objects in a range of at least 2.7% of their distance from the observer [50]. In the SPiAR study, holograms were projected at a distance of 1.5 m, thus two objects have to lie at least 4.3 cm (2.7% of 1.5 m) apart from each other in order to successfully discriminate their distance based on object size. Considering further aspects to ensure that the task is not 2D-solvable, objects were not overlapping and were projected without lighting or shadowing. Furthermore, kinetic depth cues were eliminated by anchoring the position of objects in the user’s visual field. Conclusively, true stereoscopic vision was necessary to distinguish between the distance of holograms in the SPiAR study.

The validity of the designed tasks to assess true stereoscopic vision in the AR environment is further supported by findings in the healthy population. The ability to judge distances and perceive depth in the AR environment was predicted by and correlated highly with results in clinical stereovision tests.

### Practical relevance and outlook

With a testing duration of approximately 20 min and the projection of ADL objects in the user’s near field, the design of AR tasks in the SPiAR study is closely related to a therapy session. Thus, results can be transferred to the therapeutic use of the AR system in neurorehabilitation of patients after stroke. The main finding of the SPiAR study is that post-stroke patients with impairments in VSP less accurately perceive the distance of virtual 3D objects. When applying AR in rehabilitation our findings may suggest including a test of each patient’s ability to perceive 3D objects. On basis of this test, the AR display may be personalized, for instance in the use of holographic 3D cues or monocular cues (i.e., size, lighting, shading, texture, motion perspective). However, further investigation is needed to determine whether the use of monocular cues facilitates patients with visuospatial impairments to perceive depth in the AR environment as well as which monocular cues should be used by AR software developers.

The Microsoft HoloLens® 1st Generation headset used in this experiment did not provide eye tracking. However, second generation headsets will feature the possibility to track the patients’ eye movements in real time and to record them for further inspection. This could support patients who are unable to give verbal responses to the test, by indicating which of the objects the patient attends to during the test. Also, patterns of eye movements may be indicative of, for instance, neglect or nystagmus that would have to be considered when adapting an AR display for each patient.

## Conclusions

The findings of the SPiAR study provide evidence that impairments in VSP reduce the ability to accurately perceive distances and three-dimensionality in an AR environment. For correct perception of spatial relations between objects in AR, stereovision is the key aspect of VSP in the healthy population, while spatial localization/visuoconstruction is predominant in post-stroke patients. This finding is particularly important in the sense that AR is increasingly applied for neurorehabilitative treatment with patients who show impairments in VSP.

Building the bridge from research to practice, findings of the SPiAR study are valuable for the development of AR applications in the rehabilitation of patients after a stroke. Further research is needed in order to inform the designers of AR games about cues that desire to facilitate patients with impairments in VSP to accurately perceive distance and three-dimensionality in AR environments.

## Supplementary Information

**Additional file 1. Supplementary Table:** Effects of stereovision on completion times of tasks in the Augmented Reality environment.

## Data Availability

The datasets generated and analyzed during the current study are not publicly available due to individual privacy, but are available from the corresponding author upon reasonable request.

## Abbreviations

2D: Two-dimensional
3D: Three-dimensional
3DDT: 3D Detection Task
ADL: Activities of daily living
AFCT: Alternative forced choice task
AR: Augmented Reality
HS: Healthy subjects
PMT: Perceptual matching task
PT: Position task
RDS: Random dot stereogram
SPiAR: Spatial Perception in Augmented Reality
UL: Upper limb
VOSP: Visual object and space perception
VR: Virtual reality
VSP: Visuospatial perception
