# Supplementary material for: The impact of visuospatial perception on distance judgment and depth perception in an Augmented Reality environment in patients after stroke: an exploratory study
Source: J Neuroeng Rehabil. 2021 Aug 21;18:127. doi: 10.1186/s12984-021-00920-5 (PMC8379833; doi:10.1186/s12984-021-00920-5)
Supplement: Supplementary file 1 — Additional file 1. Supplementary Table: Effects of stereovision on completion times of tasks in the Augmented Reality environment. [file 12984_2021_920_MOESM1_ESM.pdf]

Effects of stereovision on completion times of tasks in the Augmented Reality environment.

	**Patients**		**Healthy Subjects**	
	stereonormal	stereoimpaired	stereonormal	stereoimpaired
PMT	18.9 (7.7)	21.2 (14.4)	11.8 (6.4)	12.6 (5.8)
AFCT	12.6 (6.6)	11.7 (3.9)	7.8 (3.3)	6.9 (4.8)
PT	7.3 (2.6)	8.4 (3.8)	3.6 (1.6)	4.2 (1.8)
3DDT	6.0 (3.1)*	10.3 (4.8)	3.2 (1.2)*	11.1 (18.9)

Note. Cells represent *mean* (*SD*). * indicate a significant difference to the corresponding stereoimpaired group. 3DDT: 3D Detection Task, AFCT: Alternative Forced Choice Task, PMT: Perceptual Matching Task, PT: Position Task.
